# Pathogenesis and Clinical Management of Vulvovaginal Candidiasis in Mexican Diabetic Patients: A Literature Review

**DOI:** 10.7759/cureus.86012

**Published:** 2025-06-14

**Authors:** Emilio Mondragón Rosas, José Emiliano González Flores, Ana D Zamudio Carías, Nathalia García Martínez, Elisa X Díaz Salcedo, Pablo E Navarro López, Emiliano Murillo Mendoza, Michelle Cruz Méndez, Lourdes Rivas Ayala, Maria E Itami Sordo

**Affiliations:** 1 School of Medicine and Health Sciences, Tecnológico de Monterrey (ITESM), Mexico City, MEX; 2 Department of Obstetrics and Gynecology, Specialty Clinic, October 1st Ambulatory Surgery Center, Mexico City, MEX; 3 Faculty of Higher Studies Iztacala, Universidad Nacional Autónoma de México (UNAM), Mexico City, MEX

**Keywords:** antifungal resistance, candida albicans, mexico, recurrent vulvovaginal candidiasis, type 2 diabetes mellitus, vulvovaginal candidiasis

## Abstract

Vulvovaginal candidiasis (VVC) is a common fungal infection among women, with a significant subset experiencing recurrent or complicated episodes, particularly those with type 2 diabetes mellitus (T2DM). The interplay between hyperglycemia, immune dysregulation, and alterations in the vaginal microbiota creates a favorable environment for fungal persistence. Mexican women with T2DM often face more frequent and severe episodes, sometimes involving *Candida *spp. that are less responsive to standard treatments. This review examines the pathophysiological mechanisms, clinical presentation, antifungal resistance patterns, and therapeutic considerations relevant to the management of VVC in diabetic patients in Mexico. Emphasis is placed on tailored diagnostic approaches, including species-level identification through CHROMagar and MALDI-TOF, susceptibility-guided treatment, and the emerging role of next-generation antifungals such as oteseconazole. Addressing these challenges requires an integrated strategy that combines clinical vigilance with innovative diagnostic tools, evolving therapeutic options, and patient-centered education.

## Introduction and background

Vulvovaginal candidiasis (VVC) is a highly prevalent opportunistic fungal infection that affects up to 75% of women of reproductive age, with 5% to 10% developing recurrent clinical forms, characterized by more than three or four symptomatic episodes per year [1-3]. Clinically, it presents with intense vulvovaginal pruritus, burning, dyspareunia, dysuria, and irritation accompanied by thick white discharge, symptoms that significantly affect quality of life by interfering with sexual activity, daily routines, and the patient’s emotional well-being [4,5].

Among the most relevant predisposing factors for VVC are the use of broad-spectrum antibiotics, pregnancy, hormonal changes, and, notably, type 2 diabetes mellitus (T2DM) [2]. During pregnancy, elevated estrogen levels increase glycogen deposition in the vaginal epithelium, creating a carbon-rich environment that promotes the growth and adherence of *Candida* spp. [6]. This hormonal milieu, particularly in the second and third trimesters, favors a shift toward fungal overgrowth due to the associated alterations in the local microbiota and immune response [6].

The latter has been consistently identified as a significant risk factor for complicated or recurrent forms of VVC due to its effects on vaginal mucosal immunity and the balance of the microbiota [7]. Chronic hyperglycemia promotes glucosuria and increases glucose concentration in vaginal fluids, creating an ideal environment for the growth and adherence of *Candida* spp. [4,8]. In women with T2DM, vaginal colonization by *Candida albicans* strongly correlates with poor glycemic control and the presence of glucosuria, triggering symptomatic episodes of candidiasis [8]. Furthermore, diabetes leads to multifactorial immune dysfunction, including impaired activity of neutrophils and lymphocytes, as well as a diminished Th1/Th17 response, all of which facilitate fungal overgrowth [9,10]. Taken together, these mechanisms establish T2DM as a central and well-documented risk factor for recurrent and complicated VVC.

Additional factors that may predispose to VVC include the use of certain family planning methods such as intrauterine devices (IUDs) and spermicides. IUDs provide contraception primarily through local inflammatory reactions within the uterine cavity that are toxic to sperm and ova. At the same time, spermicides act by disrupting sperm membranes, thereby reducing motility and fertilization capacity. However, their use may also alter the vaginal microenvironment [3,4]. IUDs may modify the local mucosa or facilitate biofilm formation, increasing susceptibility to *Candida* colonization [6]. Spermicides like nonoxynol-9 can disrupt the normal vaginal microbiota and mucosal barrier, reducing the protective role of lactobacilli and enhancing the likelihood of fungal infection [6].

This imbalance between the fungus, local immunity, and the vaginal microbiota makes T2DM a powerful cofactor that promotes the transition of *Candida* from a commensal to a pathogenic state. A recent meta-analysis estimates that up to 68% of women with T2DM experience at least one episode of VVC, with many developing recurrences that are refractory to conventional treatments [11]. In Mexico, official records document more than 50,000 annual cases of urogenital candidiasis in the general female population aged 25 to 44 years [12]. This age group also exhibits a notable burden of T2DM, with a national prevalence of approximately 13.4% among Mexican women aged 20 years and older [13].

Over recent decades, the incidence of VVC in Mexico has declined progressively [11], partly due to public health policies, including the regulation of antibiotic use. However, this trend has plateaued in recent years. Despite the evident clinical burden, important gaps remain in the literature regarding the specific expression of this condition in Mexican women. Recurrent cases are often underestimated, and there is limited standardization of the most effective therapeutic regimens for patients with metabolic comorbidities. In this context, the present article provides a critical and comprehensive review of the available literature on VVC in women with T2DM, integrating both international evidence and the specificities of the Mexican context, with an emphasis on epidemiology, pathophysiology, antifungal resistance, clinical and microbiological diagnosis, and current treatment strategies.

This narrative review aims to synthesize the current clinical and epidemiological evidence on VVC in women with T2DM, with a particular focus on the Mexican population. By clarifying the immunometabolic interplay and therapeutic challenges specific to this region, the article aims to support clinicians in recognizing, diagnosing, and treating VVC more effectively in high-risk demographics.

## Review

Methods

This narrative review was conducted through a non-systematic synthesis of the literature published between January 2013 and April 2024. The search focused on peer-reviewed articles indexed in PubMed, Scopus, SciELO, and official institutional sources such as the Instituto Mexicano del Seguro Social (IMSS). Only full-text articles in English or Spanish were included. The sources were identified through searches in databases including PubMed, Scopus, and Google Scholar, using combinations of keywords such as “vulvovaginal candidiasis”, “*Candida albicans*”, “type 2 diabetes mellitus”, “antifungal resistance”, and “Mexico”. Articles were selected based on their relevance to the epidemiology, pathophysiology, diagnosis, treatment, and antifungal resistance patterns of VVC in women with T2DM, with an emphasis on studies involving Mexican populations. Both primary research and authoritative guidelines (e.g., IMSS, Centers for Disease Control and Prevention (CDC), Infectious Diseases Society of America (IDSA)) were included to ensure clinical applicability. No formal inclusion/exclusion criteria were applied due to the narrative nature of the review. Although a formal PRISMA protocol was not used, essential components such as source identification, thematic categorization, and relevance to clinical and microbiological aspects were included to ensure methodological transparency.

Pathogenesis of *Candida albicans* in T2DM

*Candida albicans* is a commensal fungus that forms part of the microbiota of the vagina, gastrointestinal tract, and oral cavity. Under normal conditions, its growth is kept in check by the action of beneficial bacteria such as *Lactobacillus* spp., which produce lactic acid and hydrogen peroxide, creating a hostile environment for *Candida* spp. [13]. However, under certain conditions, such as dysbiosis, prolonged antibiotic use, oral contraceptives, hormonal alterations, or changes in vaginal pH, *Candida albicans* may behave as an opportunistic pathogen [2]. The transition of *Candida albicans* from its yeast form to hyphae represents a key step in its virulence. This morphological change enhances its ability to adhere to, invade, and resist host defense mechanisms. During this transformation, the fungus activates virulence genes, including ALS3 and HWP1 (adhesins), SAPs (secreted aspartyl proteases), ECE1 (which encodes candidalysin), and BCR1 (involved in biofilm formation), which enable persistent colonization, tissue disruption, and immune evasion [2,13]. Epithelial adhesion primarily occurs through surface proteins such as Als1-Als9, Hwp1p, and Ssa1p, which bind to receptors such as E-cadherin and EGFR/Her2 on vaginal epithelial cells [2]. This facilitates both endocytosis and active fungal penetration into the epithelium.

Host immune and genetic factors in recurrent VVC

**Figure 1:**
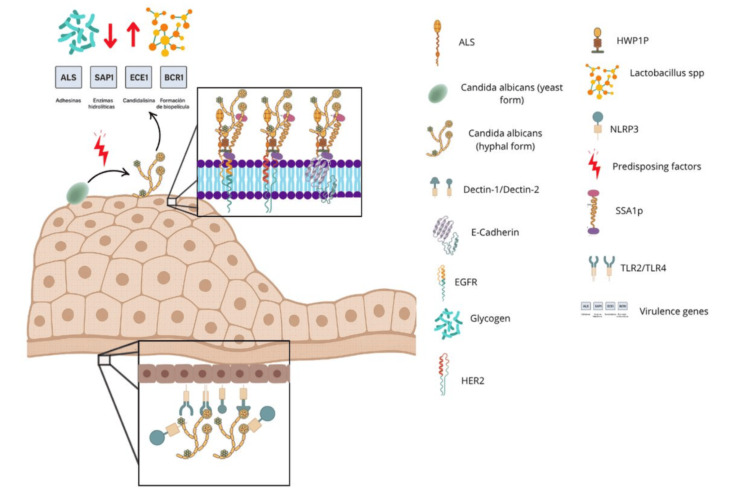
Virulence factors of Candida albicans and its interaction with the vaginal mucosa during vulvovaginal infection Adapted for illustrative purposes in the context of patients with T2DM. Illustration created by the authors. No external sources were used, and no permission is required for reproduction. T2DM: type 2 diabetes mellitus

Once inside the tissue, vaginal epithelial cells recognize the pathogen via pattern recognition receptors such as TLR2, TLR4, Dectin-1, and NLRP3. This activation triggers inflammatory pathways such as MAPK and the inflammasome, leading to the production of cytokines including IL-1β, IL-6, and TNF-α, as well as chemokines, alarmins (S100A8/A9), and antimicrobial peptides (Figure 1) [14]. This results in a massive recruitment of neutrophils, which, although part of the host defense response, are ineffective at clearing the infection and contribute to tissue damage. This uncontrolled inflammatory process is clinically manifested as pruritus, burning, dyspareunia, and leukorrhea [10,14].

In cases of recurrent VVC, in addition to environmental and behavioral factors, a genetic predisposition has been identified. This is associated with polymorphisms in genes related to innate immunity, such as TLR2, Dectin-1, NLRP3, and CARD9. These changes impact inflammasome activation and the production of key antifungal cytokines, including IL-17 and IFN-γ. Women with recurrent VVC have been shown to exhibit an immunological profile characterized by elevated IL-4 (Th2 profile) and decreased Th17 responses, which compromises the effectiveness of antifungal immunity [10,13,15]. In particular, NLRP3 polymorphisms have been associated with exaggerated inflammatory responses to *Candida* spp., which may explain recurrence in apparently immunocompetent women [15].

Epidemiology and clinical burden of VVC in diabetic women

Multiple epidemiological studies support the close interrelationship between T2DM and VVC, highlighting the clinical relevance of understanding their link in depth [16]. Women with T2DM not only have a higher incidence of VVC but also exhibit a marked tendency toward more persistent, recurrent, and difficult-to-treat forms. Recurrent VVC, clinically defined as the occurrence of four or more confirmed symptomatic episodes of VVC within a 12-month period [3,10], affects approximately 5-9% of women of reproductive age worldwide. This condition significantly impacts quality of life, and its effects are notably aggravated when it occurs alongside comorbidities such as T2DM [14]. In asymptomatic carriers of *Candida* spp., antifungal treatment is not recommended [17]. The IMSS clinical guideline explicitly states that the presence of *Candida* spp. without symptoms does not justify antifungal use, as colonization is not considered infection, and unnecessary treatment may contribute to resistance and microbiota disruption.

A series of interrelated pathogenic mechanisms may explain treatment refractoriness in some cases of VVC. First, the growing presence of non-albicans *Candida* spp. Species such as *Candida glabrata* and *Candida krusei* present a significant therapeutic challenge, as they exhibit reduced sensitivity to azoles [8,14]. This phenomenon is compounded by their capacity to form biofilms, vaginal dysbiosis induced by a hyperglycemic environment and antibiotic use, and a dysregulated immune response that, instead of containing the infection, perpetuates inflammation and symptoms [2,10,15]. Interestingly, a large cohort study in Sweden revealed a temporal inverse association between recurrent VVC and T2DM. It showed that middle-aged women with a history of recurrent VVC had a 1.4-fold increased risk of subsequently being diagnosed with T2DM compared to those without a history of VVC [16]. This risk was especially high in women over 55 years of age, suggesting that recurrent VVC may serve as an early marker of undiagnosed metabolic alterations [16]. This finding has clear clinical implications, supporting the screening for hyperglycemia or glycated hemoglobin in women with particularly persistent or refractory VVC (Table 1) [16].

**Table 1:** Prevalence of VVC in specific subgroups A large cohort study by Brieditis et al. (2024), based on longitudinal national registry data from Sweden, reported that women with a history of recurrent VVC had a 78% increased risk of subsequently being diagnosed with T2DM compared to those without VVC (HR: 1.78; 95% CI: 1.48–2.12). This was a retrospective cohort study that included over 150,000 women aged 18 to 70 years, followed for a median period of 7.1 years. The risk was especially pronounced among women over 55 years, suggesting recurrent VVC may act as a clinical marker of undetected metabolic disturbance. VVC: vulvovaginal candidiasis, T2DM: type 2 diabetes mellitus, HR: hazard ratio, CI: confidence interval, HIV: human immunodeficiency virus

Reference	Subgroups	VVC estimated prevalence
Brieditis et al., 2024 [16]	T2DM	Retrospective cohort (n >150,000): women with recurrent VVC had a 78% increased risk of T2DM diagnosis (HR: 1.78; 95% CI: 1.48-2.12)
Mohamed et al., 2022 [11]	Pregnant women	Overall pooled prevalence of VVC: 29.2%
Seeniammal et al., 2020 [18]	HIV positive women	Most common predisposing factor: HIV-induced immunosuppression (48.7%)

Antifungal resistance and treatment challenges

In most cases, VVC is caused by *Candida albicans*. However, multiple studies have documented a progressive shift in the etiological pattern of infection, especially among women with T2DM or those with a history of prolonged antifungal treatment. In this group, non-albicans species such as *Candida glabrata*, *Candida krusei*, *Candida tropicalis*, and *Candida parapsilosis* have become more prominent [8,9]. While *Candida albicans* continues to account for 85-90% of VVC cases in the general population, this percentage drops significantly among diabetic patients [8]. VVC is recognized as one of the most common fungal infections among women of reproductive age. It is estimated that 75% of women will experience at least one episode in their lifetime, and 45% of these will have two or more recurrences [1]. This scenario is particularly concerning when analyzed in high-risk subgroups.

These data highlight that, in Mexico, the burden of VVC among women with T2DM is both quantitatively significant and clinically complex. The annual documentation of over 49,000 cases in women aged 25 to 44, many of whom fall within the demographic most affected by T2DM, illustrates the convergence of two highly prevalent conditions with a synergistic impact on women's health. This intersection not only increases the risk of recurrent and refractory infections but also highlights systemic challenges, such as limited antifungal stewardship and the lack of standardized diagnostic protocols, within public healthcare systems. Furthermore, the growing predominance of *Candida* non-albicans species and rising rates of fluconazole resistance in national datasets deviate from international patterns, underscoring the urgent need for region-specific research, surveillance, and treatment guidelines. Recognizing these local epidemiological dynamics is crucial for enhancing clinical decision-making, informing public health strategies, and achieving better outcomes in a population disproportionately affected by metabolic and infectious comorbidities.

In Mexico, data from the IMSS for the period 2020-2023 indicate a concerning incidence among women over the age of 10, with 200.09 cases per 100,000 inhabitants. This condition primarily affects women between the ages of 25 and 44, with annual case numbers exceeding 49,000. According to data from the Mexican Ministry of Health, the incidence of urogenital candidiasis in this age group was 200.09 cases per 100,000 women between 2020 and 2023 [17]. In parallel, the prevalence of T2DM among Mexican women aged ≥20 years was 13.4%, with the 40-49-year subgroup showing the highest burden [13]. These overlapping age distributions underscore a high-risk population in which candidiasis and diabetes frequently coexist and compound each other’s clinical severity.

The epidemiological shift toward non-albicans species is particularly relevant, as these species often present primary or secondary resistance to azole antifungals. *Candida glabrata* and *Candida krusei*, for instance, exhibit reduced sensitivity profiles to fluconazole: *Candida krusei* is intrinsically resistant, and *Candida glabrata* can develop tolerance or secondary resistance after repeated exposure [5]. In patients with T2DM, these species are more frequent, partially explaining the therapeutic failures observed in clinical practice. U.S. clinical guidelines, such as those from the CDC and IDSA, recommend a standard treatment for recurrent VVC consisting of an induction phase with oral fluconazole (100-200 mg on days 1, 4, and 7), followed by a maintenance phase with 150 mg weekly for six months. This regimen has demonstrated remission rates of up to 90% in *Candida albicans* infections, though it is less effective against resistant or non-albicans strains [19]. In addition to fluconazole, Mexican guidelines include nystatin as an alternative for mild cases and discourage short courses in diabetic patients [17]. Resistance profiles documented by Al Halteet et al. show that *Candida krusei* exhibits complete resistance to fluconazole. At the same time, some *Candida albicans* strains display multidrug resistance, supporting the need for individualized therapy guided by antifungal susceptibility testing [8].

Diagnostic approaches in diabetic patients with VVC

The extensive and often indiscriminate use of azoles has driven the selection of resistant strains. In the last decade, multiple cases of fluconazole-resistant *Candida albicans* have been documented in women with recurrent VVC, frequently associated with prior use of the drug [5]. An epidemiological study reported that virtually all patients with fluconazole-resistant *Candida albicans* had previously received treatment with fluconazole, underscoring the role of self-medication and the availability of over-the-counter antifungals in fostering resistance [3]. Moreover, non-albicans species tend to exhibit primary resistance: *Candida glabrata* shows elevated MICs to fluconazole and may require alternative or higher-dose treatments, while *Candida krusei* is intrinsically resistant [9]. Al-Halteet et al. even identified multidrug-resistant strains of *Candida albicans* and *Candida krusei*, which is particularly alarming in contexts such as T2DM. In HIV-positive women, the IMSS guideline states that treatment protocols do not differ from immunocompetent individuals unless there are signs of complicated VVC [8,17]. However, closer monitoring is advised due to potential recurrence and coinfections [8].

Given this scenario, accurate identification of the causative species and its antifungal susceptibility profile becomes a crucial step in clinical decision-making [9]. Fortunately, technological advances in fungal diagnostics have expanded the tools available for this purpose. Although the classic clinical presentation of VVC includes vulvar pruritus, burning, erythema, and thick white discharge [4], these symptoms are nonspecific. Therefore, in women with T2DM, it is recommended to confirm the diagnosis through complementary tests before initiating prolonged treatments [8]. The diagnostic workup includes a wet mount with 10% KOH, which has a sensitivity of 40-60%, and Gram staining, with a sensitivity of around 65% [6,17]. Culture on Sabouraud dextrose agar is recommended as the gold standard, particularly in cases of recurrence or complexity. According to Al Halteet et al., CHROMagar *Candida* spp. was effective in distinguishing between species based on colony color, and identification was confirmed with the VITEK 2 system and ITS1/ITS4 gene sequencing, highlighting the reliability of phenotypic and molecular tools in species-level identification [6]. Direct microscopic examination with 10% KOH is a simple and useful tool, although its sensitivity ranges between 50% and 70%. In negative cases with high clinical suspicion, fungal culture becomes essential, particularly in recurrent episodes or patients with chronic diseases. Media such as CHROMagar *Candida* spp. enable the visual differentiation of common species. At the same time, automated systems like VITEK 2 and technologies like MALDI-TOF mass spectrometry provide rapid and accurate results for both species identification and resistance profiles [8,9]. In terms of clinical applicability, direct microscopic examination with 10% KOH and Gram staining is widely used in public healthcare settings due to its low cost (typically under USD 1 per test) and immediate availability, despite having low-to-moderate sensitivity (40-65%) and an inability to identify species. Fungal culture on Sabouraud dextrose agar, although more sensitive (up to 95%) and allowing for species identification, is less accessible in rural clinics due to longer turnaround times and the need for specialized lab infrastructure. CHROMagar *Candida* spp. improves species differentiation at a moderate cost (5-10 USD) but still requires incubation. In contrast, automated systems such as VITEK 2 or MALDI-TOF provide high specificity and sensitivity (>90%), yet their high cost and limited availability restrict their routine use in low-resource settings. Thus, in the Mexican public sector, clinicians often rely on a tiered diagnostic strategy balancing clinical suspicion, cost, and available resources.

Management strategies and novel therapeutic options

Antifungal treatment selection should consider the species identified, its susceptibility profile, and the patient’s metabolic status. In women with T2DM, even uncomplicated acute episodes require close monitoring. Traditional regimens, including oral fluconazole 150 mg as a single dose or topical azoles for three to seven days, remain effective in most cases [5,20]. However, Mexican clinical guidelines emphasize controlling the underlying metabolic disorder, avoiding short-course treatments, and including nystatin as a safe alternative [17]. For complicated VVC or recurrent VVC, more intensive regimens are recommended: fluconazole 150 mg every 72 hours for two to three doses, or alternative treatments such as voriconazole, boric acid, or nystatin ovules [1]. Strict metabolic control is essential, as improving immune function and reducing glucosuria directly lowers the fungal burden and reduces the risk of recurrence [9]. For recurrent VVC management, maintenance therapy with fluconazole 150 mg weekly for six months remains the most widely used strategy. This regimen reduces the risk of recurrence by up to 95% during the treatment period [21]. However, about half of patients relapse after discontinuation, highlighting the need for longer-lasting or innovative therapies [10]. Long-term fluconazole use is not without adverse effects, including hepatotoxicity, gastrointestinal intolerance, drug interactions, and teratogenic risk [21].

In light of these limitations, new antifungals such as oteseconazole have been developed. This next-generation triazole targets fungal lanosterol 14α-demethylase with increased specificity, minimizing off-target effects. It has demonstrated significantly lower relapse rates (5% at 48 weeks) compared to fluconazole (42%) in women with recurrent VVC [21]. Additionally, immunotherapeutic approaches, such as NDV-3A-a, a recombinant vaccine based on the Als3p adhesin from *Candida albicans*, and PEV7, based on Sap2 antigens, are under clinical evaluation in Phase II trials. These vaccines aim to stimulate robust Th1 and Th17 responses, thereby enhancing mucosal immunity and reducing the risk of recurrence. While not yet approved for clinical use, they show particular promise in immunocompromised or metabolically vulnerable populations, such as women with T2DM, due to their capacity to overcome local immune dysregulation [10].

## Conclusions

Given the substantial burden of VVC in Mexican women with T2DM, there is a pressing need for updated clinical guidelines that consider routine *Candida* spp. identification and antifungal susceptibility testing, particularly in recurrent or treatment-resistant cases. Implementing such diagnostic practices could improve therapeutic outcomes and reduce the inappropriate use of antifungals, which contributes to the development of resistance. Moreover, structural limitations within Mexico’s public health system, such as limited access to fungal culture media, molecular diagnostics, or CHROMagar in primary care settings, highlight the importance of resource-sensitive algorithms. Integrating these insights into national policy could lead to more standardized, evidence-based care and inform resource allocation for this vulnerable population.

In the context of Mexico, VVC in women with T2DM represents a complex clinical condition that reflects both local epidemiological burdens and systemic healthcare limitations. This condition results from the interaction between a chronic metabolic disorder and a recurrent opportunistic infection, and it is particularly relevant for Mexican women given the high national prevalence of T2DM and the frequent underdiagnosis of fungal infections. Hyperglycemia and immune dysregulation induced by T2DM promote persistent *Candida* spp. colonization, leading to repeated episodes that significantly impact patients’ quality of life. Moreover, the emergence of more resistant non-albicans species in this population further complicates treatment, making accurate microbiological diagnosis, including species identification and antifungal susceptibility profiling, essential.

Successful management of this condition requires a comprehensive approach that combines targeted antifungal therapy, strict glycemic control, attention to comorbidities, and continuous patient education. This personalized strategy not only reduces recurrence rates but also improves the physical and emotional well-being of a highly vulnerable population. Both patients and healthcare providers face major challenges: patients must cope with chronic symptoms and adhere to prolonged treatment regimens, while clinicians must deliver effective, empathetic, and evidence-based care.

Fortunately, advances in research have opened new therapeutic perspectives, from novel antifungal agents to emerging interventions, such as probiotics or vaginal microbiota transplantation, which may transform the future management of this condition. Integrating the latest scientific evidence with clinical experience allows physicians to guide their patients toward more effective control of VVC. Ultimately, addressing this condition requires understanding it from both a medical and psychosocial perspective: a balance between the rigor of treatment and empathy for what it means to live with both diseases.
